# Two-Degree-of-Freedom Piezoelectric Energy Harvesting from Vortex-Induced Vibration

**DOI:** 10.3390/mi13111936

**Published:** 2022-11-09

**Authors:** De Lu, Zhiqing Li, Guobiao Hu, Bo Zhou, Yaowen Yang, Guiyong Zhang

**Affiliations:** 1 State Key Laboratory of Structural Analysis for Industrial Equipment, Dalian University of Technology, Dalian 116000, China; 2 School of Civil and Environmental Engineering, Nanyang Technological University, Singapore 639798, Singapore

**Keywords:** piezoelectric, vortex-induced vibration, wind energy harvesting, two-degree-of-freedom

## Abstract

In recent years, vortex-induced vibration (VIV) has been widely employed to collect small-scale wind energy as a renewable energy source for microelectronics and wireless sensors. In this paper, a two-degree-of-freedom (2DOF) VIV-based piezoelectric energy harvester (VIVPEH) was designed, and its aerodynamic characteristics were thoroughly investigated. First, based on the traditional model theory and combined with the knowledge of vibration dynamics, the governing equations of the 2DOF VIVPEH were established. The dynamic responses, including the displacement and voltage output, were numerically simulated. Compared with the traditional 1DOF VIVPEH, the 2DOF VIVPEH proposed in this paper produced two lock-in regions for broadband wind energy harvesting. Furthermore, it was unveiled that the first- and second-order resonances were induced in the first and lock-in regions, respectively. Subsequently, a parametric study was conducted to investigate the influences of the circuit and mechanical parameters on the energy harvesting performance of the 2DOF VIVPEH. It was found that when the 2DOF VIVPEH was induced to vibrate in different lock-in regions, its optimal resistance became different. Moreover, by varying the masses and stiffnesses of the primary and secondary DOFs, we could adjust the lock-in regions in terms of their bandwidths, locations, and amplitudes, which provides a possibility for further customization and optimization.

## 1. Introduction

In recent years, integrated circuits, microsensors, and other low-power consumption electronic products have been swiftly developed and widely implemented in various engineering applications. However, the disadvantages of using chemical batteries to provide a power supply have been gradually exposed, such as toxicity, risk of causing environmental pollution, limited lifespan, and the need for frequent replacement [1]. Hence, a sustainable energy supply technology is needed. In the context of the increasing awareness of energy saving, the emission reduction in carbon dioxides, and environmental protection, researchers have proposed to harvest renewable energy from the ambient environment [2,3]. Environmental vibration can generally be classified into two categories: (1) concomitant excitations generated during machinery operation, vehicle movements, or human motion, and (2) flow-induced vibration generated by aeroelastic instability, such as vortex-induced vibration (VIV) [4], galloping [5,6,7], fluttering [8,9], buffeting [10], or their synergy [11,12]. VIV has attracted lots of research interest due to its relatively low cut-in wind speed.

Researchers have developed various VIV-based piezoelectric energy harvesters (VIVPEHs) and conducted many studies on VIV energy harvesting. Taylor et al. [13] designed an eel-like device that uses periodic eddy currents generated behind a bluff body to make the piezoelectric plate vibrate for producing a power output. Song et al. [14] used a piezoelectric beam attached with a bluff body to convert flow energy into electricity. It was shown that the output power varied with the load resistance R and flow velocity U. The increase in the maximum output power and the corresponding optimal speed were different under different configurations of the cylinder diameter and mass. Wang et al. [15] proposed an equivalent circuit model (ECM) for a typical VIVPEH. In this study, the Scanlan–Ehsan aerodynamic force model [16] was used to describe the interaction between fluid and structure, and the reliability of the model was verified using experiments. Meanwhile, researchers conducted lots of theoretical and experimental studies to improve the performance of VIVEPHs. Dai et al. [17] developed a distributed parameter model of VIVPEH and optimized the parameters to reduce the cut-in wind speed. Azadeh-Ranjba et al. [18] found that lowering the aspect ratio of a finite-length cylinder broadens the “lock-in” region of VIV and increases the vibration amplitude of the system, thereby improving the broadband energy harvesting capability.

Improving the power output and reducing the cut-in wind speed are the two main concerns in designing a galloping piezoelectric energy harvester (GPEH). All the wind energy harvesters mentioned above are one-degree-of-freedom (1DOF) designs. Analyses [19,20] show that a straightforward method to reduce the cut-in wind speed of a 1DOF GPEH is to directly reduce the mechanical damping, which is usually beyond the control of a given transduction mechanism. Therefore, researchers have proposed various innovative configurations to improve the performance of GPEHs. Tang et al. [21] proposed a nonlinear energy harvester with a magnetic oscillator. The introduction of the magnetic oscillator not only enlarged the bandwidth but also greatly increased the power output magnitude. Wu et al. [22] incorporated the magnetic nonlinearity into a 2DOF system. They found that by appropriately adjusting the distance between the magnets, the first two resonant peaks could be tuned close to each other to obtain a wide operational bandwidth. Su et al. [23] found that a bistable 2DOF VEH could exhibit inter-well and chaotic oscillations, which may benefit broadband energy harvesting.

The method of introducing parasitic beams to increase the degree of freedom of the system is a new research direction in recent years. The main analysis objects are basically galloping piezoelectric energy harvesters. Zhao et al. [24] developed a 2DOF GPEH with nonlinear magnetic interaction based on a cut-out beam structure. Lan et al. [20] investigated the dynamics and performance of a 2DOF GPEH. The results showed that the 2DOF GPEH could significantly reduce the cut-in wind speed and improve the output power. Hu et al. [25] designed a physical model of a 2DOF GPEH with a secondary beam mounted on the main piezoelectric cantilever beam and evaluated the performance of the 2DOF system through experiments. Hu et al. [26] also proposed a comb-like beam-based multiple-degree-of-freedom (MDOF) piezoelectric system for harvesting wind energy by exploiting the galloping mechanism. The comb-like beam-based GPEH consists of a series of parasitic beams mounted on a conventional cantilever beam bonded with a piezoelectric transducer at the clamped end. The results showed that it outperformed the conventional GPEH in reducing the cut-in wind speed from 2.24 m/s to 1.96 m/s and enhancing the power output by about 171.2% under a specific wind speed of 3 m/s. According to the above literature review, it can be seen that adding parasitic beams to traditional cantilever beams is a good scheme for structural optimization. However, this method has not been applied to designing any VIVPEH at present.

This paper proposes a 2DOF VIVPEH system for the first time and investigates its aerodynamic characteristics as well as the energy harvesting performance achieved by the attached piezoelectric transducer. The content of this paper is organized as follows. Section 2 presents the lumped parameter model of the proposed 2DOF VIVPEH. Section 3 presents the numerical calculation results and the analyses of the aerodynamic characteristics of the proposed 2DOF VIVPEH. Based on the theoretical model, a parametric study was conducted in Section 4 to investigate the effects of several system parameters on the wind energy harvesting performance. Section 5 summarizes the conclusions obtained from this work.

## 2. Wake Oscillator Model for VIVPEH

**Figure 1:**
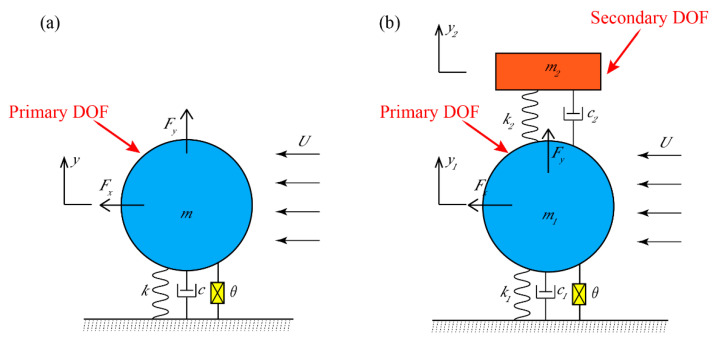
(**a**) Conventional 1DOF VIVPEH; (**b**) 2DOF VIVPEH.

**Figure 2:**
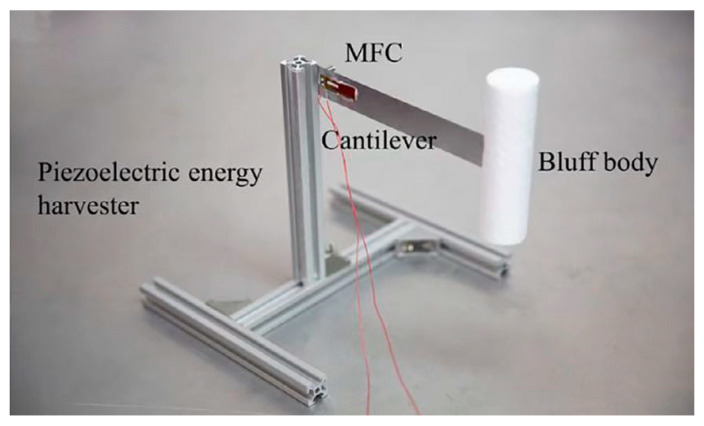
Physical prototype of a conventional 1DOF VIVPEH [15].

Inspired by previous research [15,20], this paper proposes a 2DOF VIVPEH. Figure 1a,b shows the lumped parameter models of the conventional 1DOF and our proposed 2DOF VIVPEH, respectively. Figure 2 displays the physical prototype of a conventional 1DOF VIVPEH designed by Wang et al. [15]. The 1DOF VIVPEH consists of an elastically mounted cylinder bluff body and a piezoelectric transducer. When the wind flows through, the vortices periodically shed off at the trailing edge of the bluff body, causing vortex-induced force on the bluff body and stimulating its vibration. The system will oscillate when the vortex shedding frequency is close to the natural frequency. At this time, the vibration frequency will be locked at/around the resonant frequency within a specific wind speed range, which is referred to as the “lock-in” region. Unlike the galloping phenomenon, the vibration magnitude of VIV does not monotonically increase with the wind speed. This feature can protect the system from damage due to excessively large deformation when the wind speed is too high. The 2DOF VIVPEH contains another DOF. We assume the auxiliary DOF has a much smaller geometric dimension, and the aerodynamic force applied to it is inappreciable. *m*, *k*, and *c* are the effective mass, stiffness, and damping coefficient, respectively. *y* denotes the vertical displacement. The subscripts 1 and 2 denote the two DOFs. *θ* represents the electromechanical coupling efficiency. *F_x_* and *F_y_* are, respectively, the drag and lift force on the bluff body under the action of airflow.

### 2.1. 1DOF VIVPEH

The conventional 1DOF VIVPEH, as shown in Figure 1a, has been extensively investigated by researchers. Its governing equations are briefly revisited as we will compare its performance with our proposed 2DOF VIVPEH later. The cylindrical bluff body oscillates in the Y direction under the aerodynamic force at the constant wind speed *U*. Based on the constitutive equations of linear piezoelectric materials and Euler’s beam theory [27], researchers have developed a variety of theoretical models to describe the dynamics of the 1DOF VIVPEH. The wake oscillator model is used in this paper. By describing the wake motion as a van der Pol oscillator, the theoretical model of the VIV system is established. The dynamic governing equation of the 1DOF VIVPEH can be written as [28]:

(1) $$m\ddot{y}\left(t\right)+\left[c+\frac{1}{2}C_{D}\rho DUL\right]\dot{y}\left(t\right)+ky\left(t\right)+\theta V\left(t\right)=\frac{1}{4}C_{L_{0}}\rho DU^{2}Lq\left(t\right)$$

where *y*(*t*) is the vertical displacement of the bluff body, *V*(*t*) is the voltage generated by the piezoelectric transducer, and *C_D_* and *C*_*L*0_ are the mean drag coefficient and the amplitude of the fluctuating lift force coefficient, respectively. *ρ* is the air density. *D* and *L* are the geometric dimensions of the cylindrical bluff body. q(t) is the variable to describe the motion of the near wake generated behind the bluff body. It can be deemed as an imagined variable related to the averaged transverse component of the flow [29] or an assumed variable proportional to the transverse velocity of the near-wake fluid [30]. The governing equation of q(t) follows a similar form as that of the van der Pol oscillator:

(2) $$\ddot{q}\left(t\right)+\lambda \omega_{s}\left[q^{2}\left(t\right)-1\right]\dot{q}\left(t\right)+\omega_{s}{}^{2}q\left(t\right)=\frac{A}{D}\ddot{y}\left(t\right)$$

where *λ* and *A* can be determined experimentally, which are equal to 0.3 and 12, respectively [31]. *ω_s_* is the vortex shedding frequency:

(3) $$\omega_{s}=\frac{2\pi S_{t}u}{D}$$

Assuming that the piezoelectric transducer is shunted to a simple resistive load *R_L_*, the circuit equation can then be written as:

(4) $$\frac{V\left(t\right)}{R_{L}}+C_{P}\dot{V}\left(t\right)-\theta \dot{y}\left(t\right)=0$$

where *C_p_* is the clamped capacitance of the piezoelectric transducer.

### 2.2. 2DOF VIVPEH

As aforementioned, we assume the bluff body is attached to the primary DOF and suffers the aerodynamic force, while the aerodynamic force applied to the secondary DOF is negligible. The governing equations of 2DOF VIVPEH, as shown in Figure 1b, can be written as:

(5) $$m_{1}{\ddot{y}}_{1}\left(t\right)+\left[c_{1}+c_{2}+\left(\frac{1}{2}C_{D}\rho DUL\right)\right]{\dot{y}}_{1}\left(t\right)+(k_{1}+k_{2})y_{1}\left(t\right)+\theta V\left(t\right)-c_{2}{\dot{y}}_{2}\left(t\right)-k_{2}y_{2}\left(t\right)=\frac{1}{4}C_{L_{0}}\rho DU^{2}Lq$$

(6) $$m_{2}{\ddot{y}}_{2}\left(t\right)+c_{2}\left({\dot{y}}_{2}\left(t\right)-{\dot{y}}_{1}\left(t\right)\right)+k_{2}\left(y_{2}\left(t\right)-y_{1}\left(t\right)\right)=0$$

(7) $$\frac{V\left(t\right)}{R_{L}}+C_{P}\dot{V}\left(t\right)-\theta \dot{x}=0$$

(8) $$\ddot{q}\left(t\right)+\lambda \omega_{s}\left[q^{2}\left(t\right)-1\right]\dot{q}\left(t\right)+\omega_{s}{}^{2}q\left(t\right)=\frac{A}{D}{\ddot{y}}_{1}\left(t\right)$$

The definitions of the physical parameters are consistent with those of the 1DOF VIVPEH, where subscripts 1 and 2 represent the primary DOF (i.e., the one attached with the cylindrical bluff body) and the secondary DOF, respectively.

## 3. Numerical Simulations

In this section, the performance of the 2DOF system will be evaluated using numerical simulations.

### 3.1. Numerical Method

In order to numerically solve the governing equations, Equations (5)–(8) in Section 2 were transformed into the state space form:

(9) $$\left[\begin{array}{c} {\dot{y}}_{1}\\ {\ddot{y}}_{1}\\ {\dot{y}}_{2}\\ {\ddot{y}}_{2}\\ \dot{\begin{array}{c} V\\ \dot{q}\\ \ddot{q} \end{array}} \end{array}\right]=B\cdot \left[\begin{array}{c} y_{1}\\ {\dot{y}}_{1}\\ y_{2}\\ {\dot{y}}_{2}\\ V\\ q\\ \dot{q} \end{array}\right]$$

$$B=\left[\begin{array}{ccccccc} 0 & 1 & 0 & 0 & 0 & 0 & 0\\ -\frac{\left(k_{1}+k_{2}\right)}{m_{1}} & -\frac{c_{1}+c_{2}+C_{a1}}{m_{1}} & \frac{k_{2}}{m_{1}} & \frac{c_{2}}{m_{1}} & -\frac{\theta }{m_{1}} & \frac{C_{a2}}{m_{1}} & 0\\ 0 & 0 & 0 & 1 & 0 & 0 & 0\\ \frac{k_{2}}{m_{2}} & \frac{c_{2}}{m_{2}} & -\frac{k_{2}}{m_{2}} & -\frac{c_{2}}{m_{2}} & 0 & 0 & 0\\ 0 & \frac{\theta }{C_{P}} & 0 & 0 & -\frac{1}{C_{P}\cdot R_{L}} & 0 & 0\\ 0 & 0 & 0 & 0 & 0 & 0 & 1\\ -\frac{A}{D}\cdot \frac{k_{1}+k_{2}}{m_{1}} & -\frac{A}{D}\cdot \frac{c_{1}+c_{2}+C_{a1}}{m_{1}} & \frac{A}{D}\cdot \frac{k_{2}}{m_{1}} & \frac{A}{D}\cdot \frac{c_{2}}{m_{1}} & -\frac{A}{D}\cdot \frac{\theta }{m_{1}} & -\omega_{s}^{2}+\frac{A}{D}\cdot \frac{C_{a2}}{m_{1}} & -\lambda \omega_{s}\left[q^{2}-1\right] \end{array}\right]$$

where *C_a_*_1_ and *C_a_*_2_ are expressed as:

(10) $$C_{a1}=\frac{1}{2}C_{D}\rho DUL$$

(11) $$C_{a2}=\frac{1}{4}C_{L_{0}}\rho DU^{2}L$$

The aerodynamic lift force can be obtained from (10) as *C_a_*_2_·*q* and the aerodynamics drag force as *C_a_*_1_·$\dot{y}$. In this way, the matrix differential equation can be analyzed and solved using the Runge–Kutta method. The MATLAB Ode45 function adopts the Runge–Kutta method with an adaptive step size control strategy. Based on the experimental data of the 1DOF VIVPEH model from the literature [15,28] and the parameters of an example MDOF GPEH model in the references [20,26], the masses, damping coefficients, stiffnesses, and piezoelectric properties for the 2DOF VIVPEH under investigation in this paper are listed in Table 1. The model proposed in this paper represents a conceptual design. To avoid making the conceptual design too arbitrary and unrealistic, the system parameters, including the equivalent masses, stiffnesses, damping ratios, and electromechanical coupling factor, are determined by referring to a 2DOF GPEH model [26]. Regarding the wake oscillator model, we assume the working condition is similar to that in [28] and adopt those coefficients in the equation from that article. According to the parameters listed in Table 1, the first- and second-order natural frequencies of the 2DOF VIVPEH under free undamped vibration were calculated to be 4.645 Hz and 10.292 Hz, respectively. In the simulation, the initial condition for Equation (9) was set at [0.0001 0 0 0 0 0 0]^T^, which meant the initial displacement of the cylindrical bluff body was 0.1 mm (only used to initiate the vibration).

### 3.2. Preliminary Numerical Results

The initial wind speed was selected as 4 m/s with the whole structure stationary and the bluff body in equilibrium. Under these conditions, the numerical calculation was carried out.

**Figure 3:**
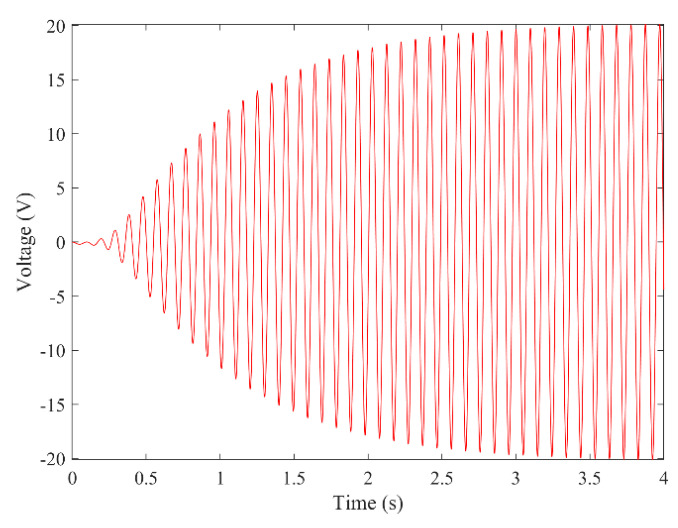
Time history voltage response (*U* = 4.0 m/s).

**Figure 4:**
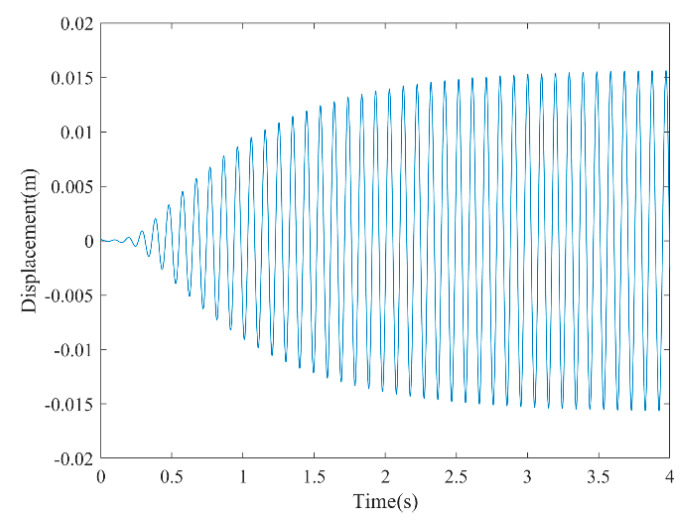
Time history displacement response (*U* = 4.0 m/s).

**Figure 5:**
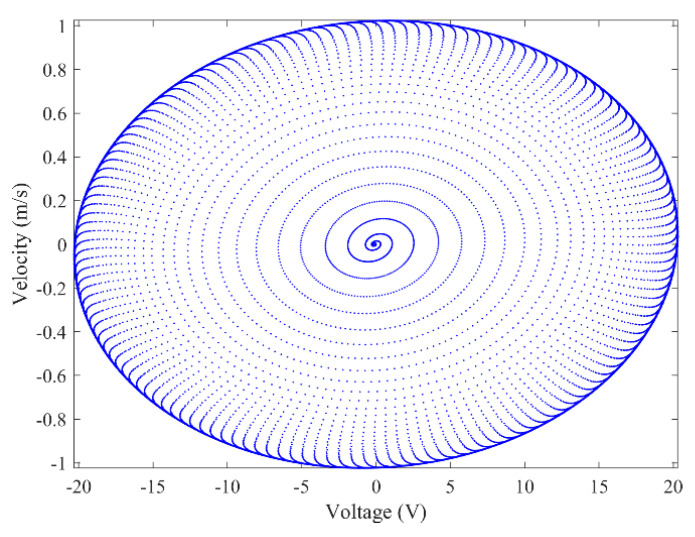
Phase portrait (limit cycle oscillation) (*U* = 4.0 m/s).

**Figure 6:**
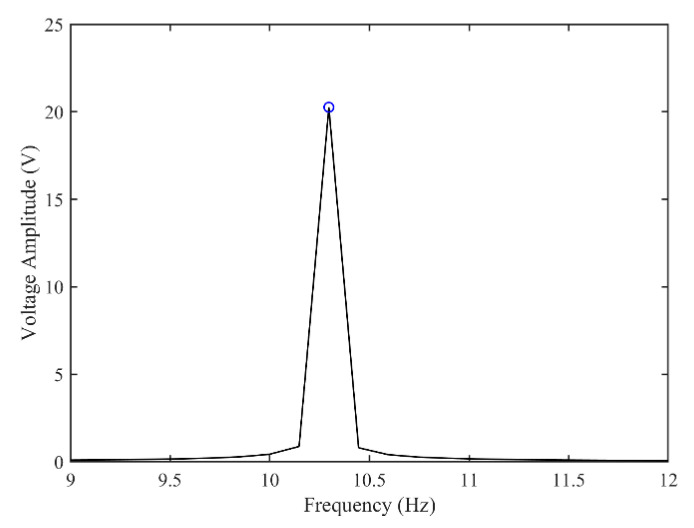
Fast Fourier transform (FFT) of the steady-state part of the voltage–time response. (*U* = 4.0 m/s).

**Figure 7:**
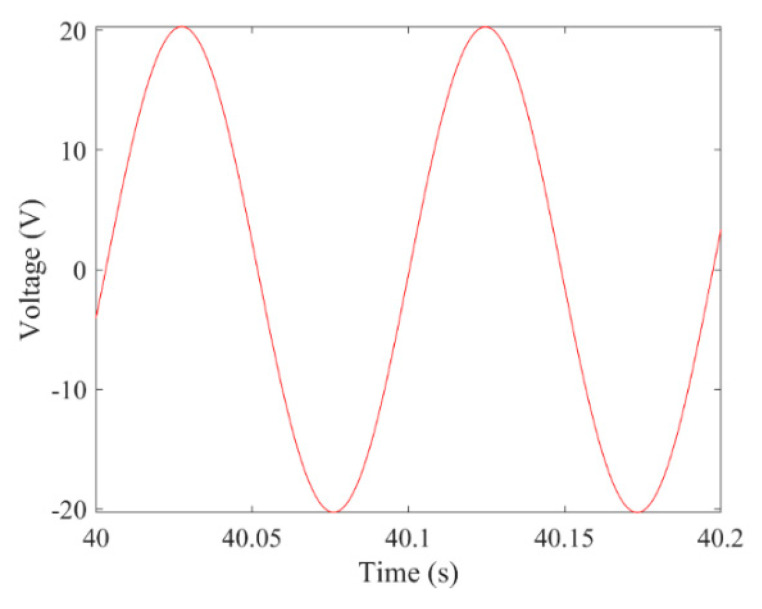
Enlarged view of the time history voltage response over two cycles.

Figure 3 and Figure 4 show the time–history responses of the voltage output and bluff body displacement, respectively. Figure 3 shows the whole process of the output voltage generation, development, and evolution. The system reaches stability at about 3.5 s, and the peak output voltage does not increase with time. The voltage and displacement evolution trends are the same: the structure begins to vibrate under the wind load, and the system reaches stability when the maximum voltage and bluff body displacement are achieved. At 4 m/s speed, the maximum voltage magnitude is 20.296 V, and the maximum displacement of the bluff body is 15.8 mm. Figure 5 shows the phase portrait of the bluff body velocity against the voltage output. One can note that the trajectory starts from the center of the graph and gradually spirals into the dark blue outer ring, which is the limit cycle. The trajectory is attracted to this limit cycle with time, which indicates the limit cycle is stable. Figure 6 is the frequency domain response diagram of the voltage obtained by the fast Fourier transform (FFT). It can be noted that the frequency spectrum is pretty clean, and there is only a single peak located at the frequency of 10.296 Hz, which corresponds to the second-order natural frequency of the 2DOF VIVPEH. Figure 7 shows the partial details of Figure 3 between 40 s and 40.2 s. It can be seen that the output voltage fluctuation trend can be approximated as sinusoidal fluctuation after the system reaches stability.

### 3.3. Aerodynamic Characteristics

On the premise of fixing other parameters, we changed the input wind speed and recorded the variation of the output voltage. According to the voltage-wind speed curve, the aerodynamic performance of the 2DOF VIVPEH was analyzed.

**Figure 8:**
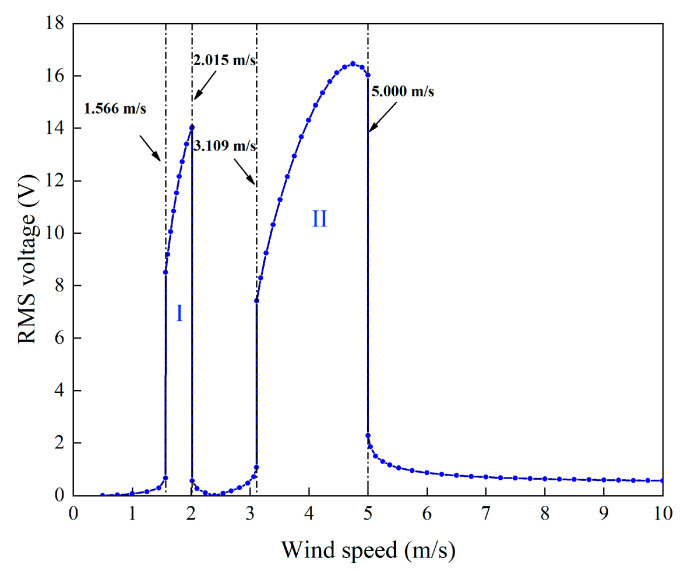
Root-mean-square (RMS) voltage versus wind speed.

It can be seen in Figure 7 that the voltage response of the 2DOF VIVPEH after reaching stable is harmonic (sinusoidal). Hence, the root-mean-square (RMS) voltage is calculated by multiplying the peak voltage with $\sqrt{2}/2$. Figure 8 presents the RMS voltage of the 2DOF VIVPEH versus the wind speed. Table 2 lists the RMS voltage outputs corresponding to several special wind speeds where sudden changes take place.

Near these typical wind speeds, even small changes in wind speed will cause significant changes in the output voltage. When the wind speed *U* = 1.566 m/s, the system output voltage sharply increases. This wind speed is called “cut-in wind speed”. Over a certain wind speed range, when the vortex shedding frequency is near the structural natural frequency, the structure reacts to the vortex shedding formation. Due to the interaction with the wake vortex shedding, vortex-induced structural resonance occurs. This effect is referred to as the lock-in phenomenon of eddy vibration, and the corresponding wind speed range is defined as the lock-in region. One can easily observe two convex regions in Figure 8. The two regions are labeled I and II, respectively. The voltage outputs over regions I and II are significantly larger than the exterior. Hence, these two regions are the “lock-in” regions of the system. A traditional 1DOF VIVPEH only has one lock-in region. Interestingly, it was found that the proposed 2DOF VIVPEH exhibits two lock-in regions.

**Figure 9:**
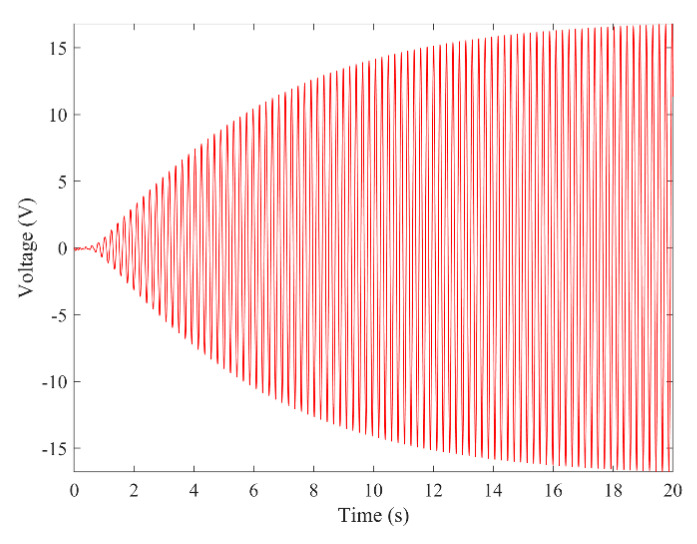
Time–history voltage response (*U* = 1.8 m/s).

**Figure 10:**
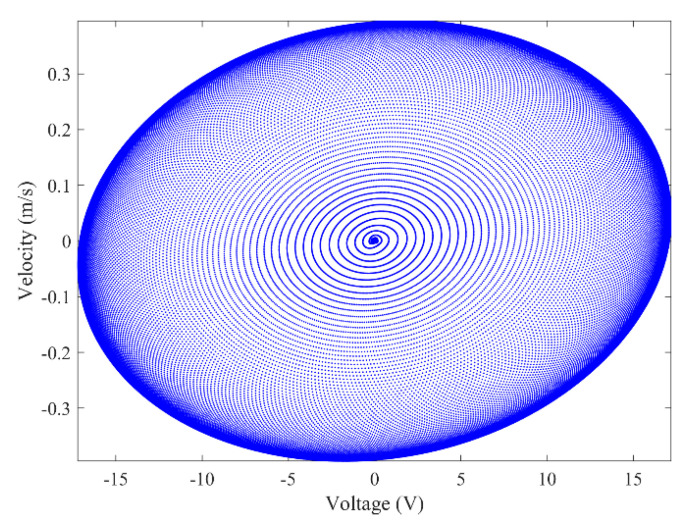
Phase portrait (limit cycle oscillation) (*U* = 1.8 m/s).

**Figure 11:**
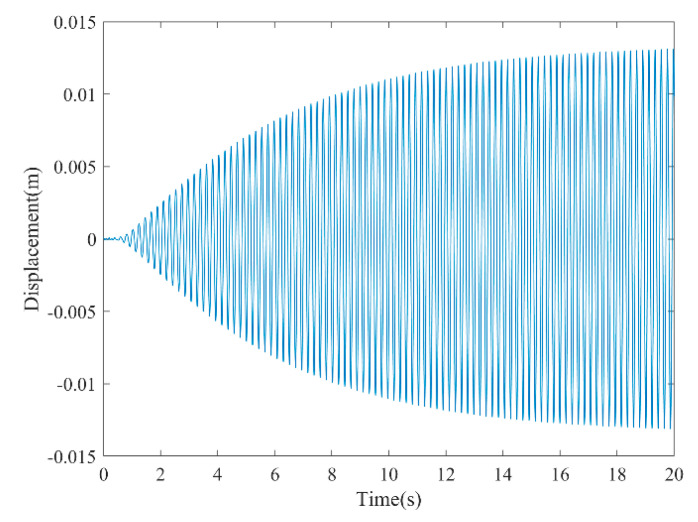
Time–history displacement response (*U* = 1.8 m/s).

**Figure 12:**
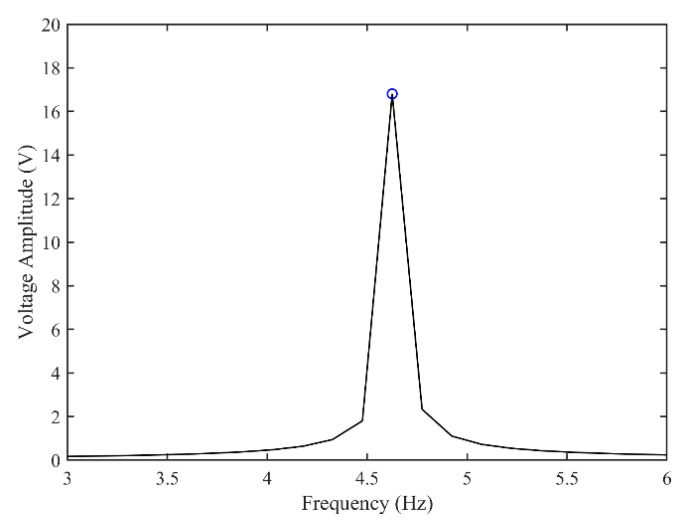
Fast Fourier transform (FFT) of the steady-state part of the voltage–time response. (*U* = 1.8 m/s).

According to Figure 7, it is known that *U* = 4.0 m/s, i.e., the wind speed of the case shown in Figure 3, Figure 4, Figure 5 and Figure 6 falls into region II. Figure 9, Figure 10, Figure 11 and Figure 12 present the results of a counterpart case from region I at the wind speed of 1.80 m/s. Compared with Figure 3, Figure 4, Figure 5 and Figure 6, it can be seen that the evolution trends of voltage and displacement are basically consistent, except the time to reach the stable state is prolonged. In the voltage frequency domain response diagram of *U* = 1.8 m/s, the peak frequency is about 4.651 Hz, which is smaller than that of the case in Figure 2 and corresponds to the first-order natural frequency of the 2DOF VIVPEH. Based on the results presented in Figure 6 and Figure 12, we can speculate that the first- and second-order resonances of the 2DOF VIVPEH are, respectively, induced in region I and II.

**Figure 13:**
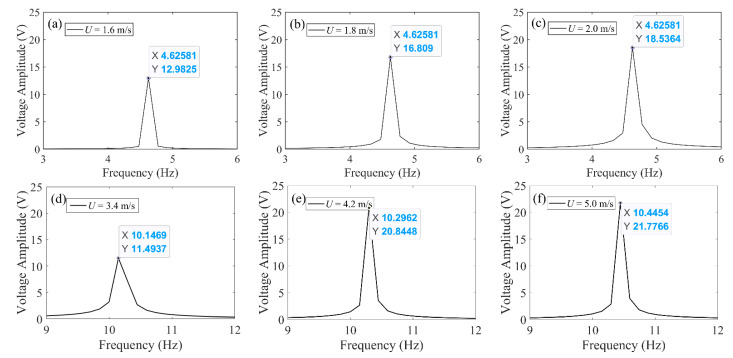
Fast Fourier transform (FFT) of the steady-state part of the voltage–time response under different wind speeds: (**a**) 1.6 m/s, (**b**) 1.8 m/s, (**c**) 2.0 m/s, (**d**) 3.4 m/s, (**e**) 4.2 m/s, and (**f**) 5.0 m/s.

In order to verify the above speculation, two sets of wind speeds, respectively, from region I and region II were selected for numerical calculation. The first row of Figure 13 presents the cases from region I. It was found that regardless of the wind speed change, the dominant frequency is always around 4.645 Hz, which is the first-order resonant frequency of the 2DOF VIVPEH. The second row of Figure 13 illustrates the cases from region II. As expected, the peak frequencies are all around 10.292 Hz, which is the second-order resonant frequency of the 2DOF VIVPEH. The slight difference between them originates from the variation of the effective damping coefficient.

## 4. Parametric Study

In this section, the influences of the system parameters on the formation of the lock-in regions and the output power are investigated to provide guidelines on properly adjusting the 2DOF VIVPEH for superior performance. The parameters to be studied are classified into two categories: circuit parameters (load resistance) and mechanical parameters (mass, stiffness, damping, etc.). Other parameters used in the analyses remain the same as those listed in Table 1.

The research on the electromechanical coupling coefficient versus the energy capture characteristic is not deeply studied in this paper. However, what is certain is that a larger electromechanical coupling coefficient indicates a stronger energy conversion ability of the piezoelectric transducer. In the weak coupling domain, increasing the electromechanical coupling coefficient will lead to an increase in the output power. In terms of the influences on aerodynamic behavior, we can easily infer that as the electromechanical coupling coefficient increases, the electrical damping increases; thus, the cut-in wind speed will increase. In fact, the electromechanical coupling coefficient affects many things. Recent papers presented comprehensive studies on this topic for a 1DOF GPEH [32,33]. The 2DOF VIVPEH is more complicated; thus, this kind of study may be a prospective work.

### 4.1. Effects of Circuit Parameters

**Figure 14:**
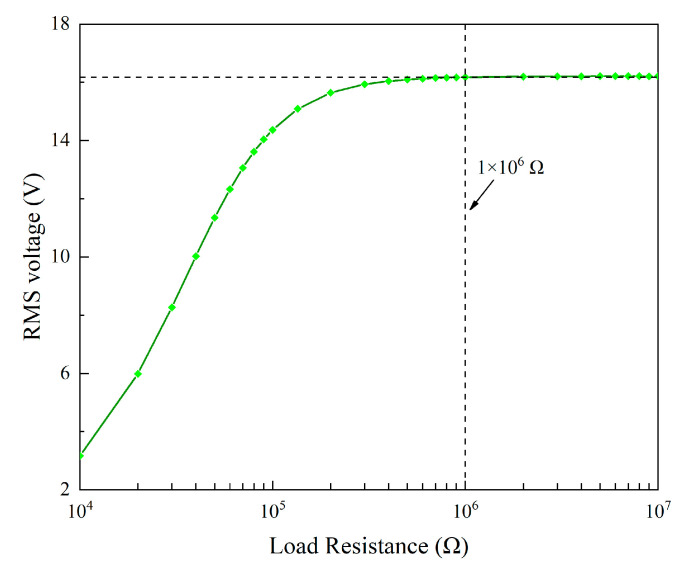
RMS voltage varies with the load resistance.

In analyzing the effect of circuit parameters, we considered a constant wind speed of 4.5 m/s. By changing the load resistance and fixing other parameters, Figure 14 presents the variation of the RMS voltage. It can be seen that the RMS voltage monotonically increases with the increase in the load resistance. When the load resistance exceeds 1 MΩ, the RMS voltage very slowly increases. This indicates that 1 MΩ is much larger than the internal impedance, and the piezoelectric transducer is almost under the open-circuit condition. Therefore, the RMS voltage approaches the open-circuit RMS voltage.

The average power is calculated as:

(12) $$P_{avg}=V_{RMS}^{2}/R_{L}$$

**Figure 15:**
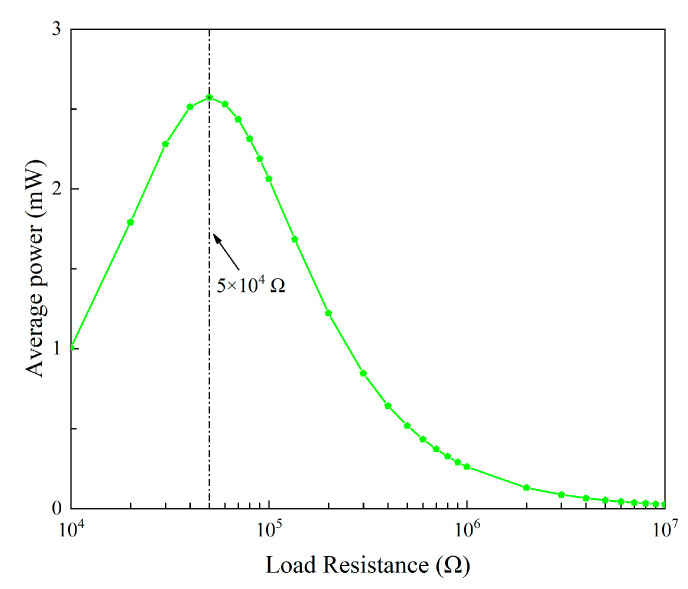
Average power varies with the load resistance.

**Figure 16:**
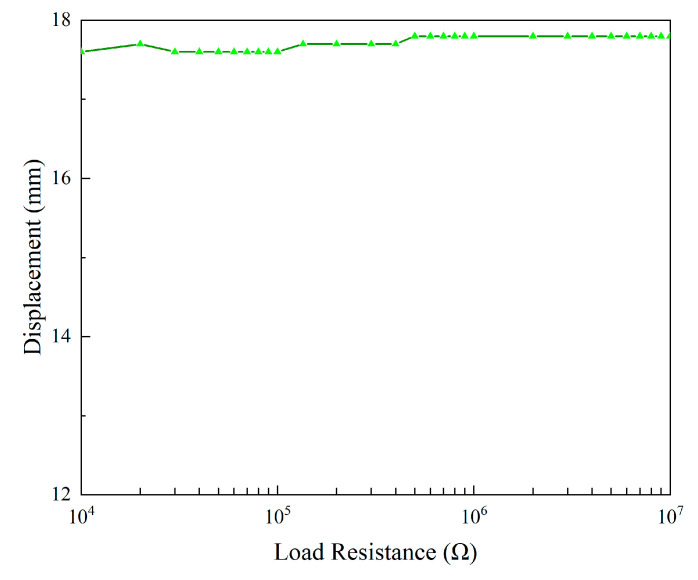
Displacement versus the load resistance.

The variation trend of the average power, as shown in Figure 15, is different from that of the RMS voltage. With the increase in the load resistance, the output power first increases and then decreases. It reaches the peak value around 5 × 10^4^ Ω. Hence, it is known that 5 × 10^4^ Ω is the optimal resistance of the 2DOF VIVPEH. As there is only a single peak in the power plot, it can be inferred that this 2DOF VIVPEH belongs to a weakly coupled piezoelectric system [33]. In other words, the change in the circuit parameters will not bring any significant influence on the dynamics of the mechanical structure. Figure 16 illustrates the effect of the load resistance on the bluff body displacement magnitude of the 2DOF VIVPEH. As predicted by the coupling strength theory, the variation of the bluff body displacement magnitude is minor and negligible in response to the change in the load resistance.

**Figure 17:**
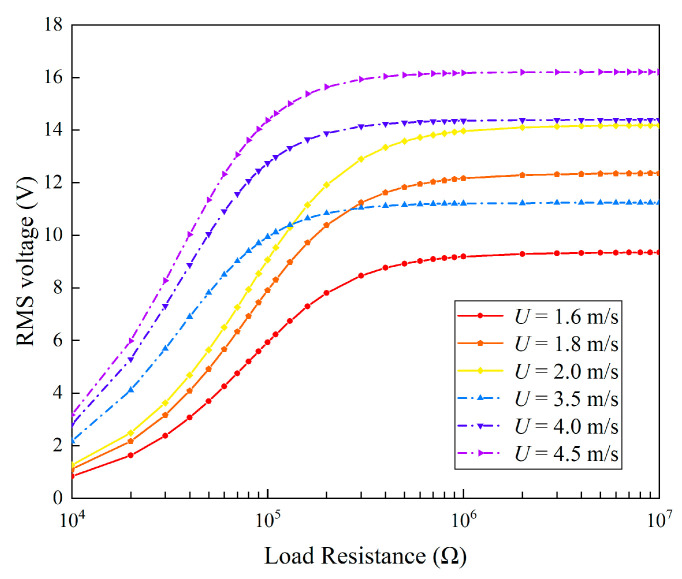
Variation of RMS voltage with load resistance at different wind speeds.

**Figure 18:**
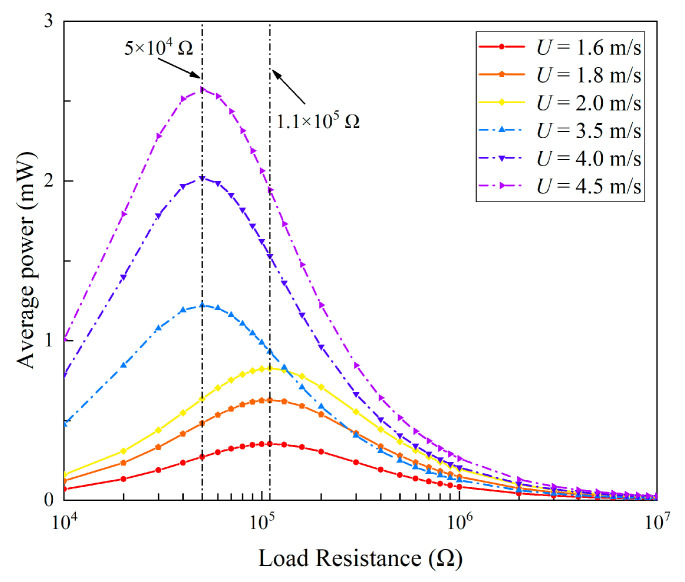
Variation of average power with load resistance at different wind speeds.

We further selected three wind speeds from the two lock-in regions (1.6 m/s, 1.8 m/s, and 2 m/s from region I; 3.5 m/s, 4 m/s, and 4.5 m/s from region II) and then changed the load resistance to examine the influences on the responses in different lock-in regions. The corresponding results are presented in Figure 17 and Figure 18. In terms of the RMS voltage output, the results are similar: the RMS voltage shows a monotonic increase trend with the increase in the load resistance. Moreover, it was noted that the 2DOF VIVPEH can more easily produce larger RMS voltage outputs when the wind speed enters the lock-in region II.

Regarding the average power shown in Figure 18, one finds that the evolution trends of the curves are similar: the maximum power is attained only when the load resistance is tuned to the optimal value. It is worth emphasizing that the optimal load resistances of the 2DOF VIVPEHs are different when the wind speed is changed to different values. One can note that when *U* = 1.6 m/s, 1.8 m/s, and 2 m/s, the optimal load resistance is 1.1 × 10^5^ Ω. When *U* = 3.5 m/s, 4 m/s, and 4.5 m/s, the optimal load resistance becomes 5 × 10^4^ Ω. This is because the excitation modes corresponding to region I and region II are different. According to the well-known formula to estimate the optimal resistance, i.e., *R*_opt_ = 1/(*ω*_n_*C_p_*), the optimal resistances corresponding to the different modes are different. Moreover, the optimal resistance for the higher-order mode should be smaller. That explains why the optimal resistance in region II is smaller.

### 4.2. Effect of Mechanical Parameters

**Figure 19:**
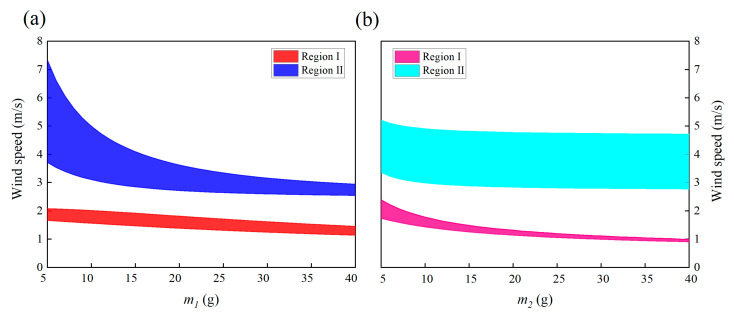
Effects of masses on the “lock-in” regions of the 2DOF VIVPEH: (**a**) *m*_1_ and (**b**) *m*_2_.

**Figure 20:**
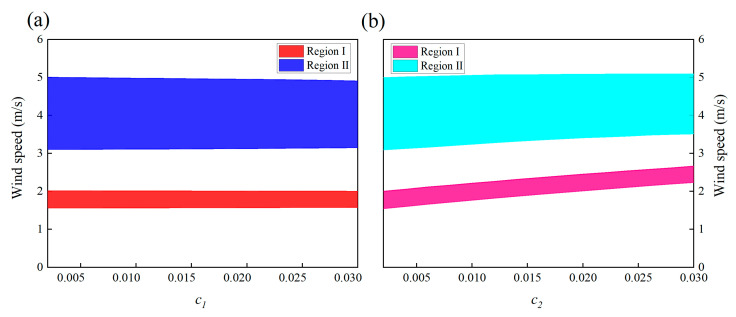
Effects of damping coefficients on the “lock-in” regions of the 2DOF VIVPEH: (**a**) *c*_1_ and (**b**) *c*_2_.

**Figure 21:**
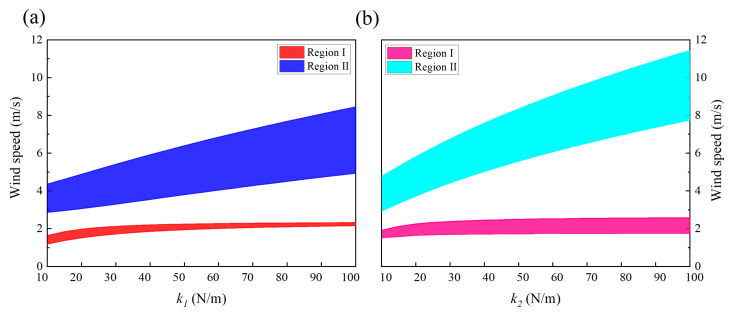
Effects of stiffnesses on the “lock-in” regions of the 2DOF VIVPEH: (**a**) *k*_1_ and (**b**) *k*_2_.

This section discusses the effects of several mechanical parameters, including masses, stiffnesses, and damping coefficients on the energy harvesting performance of the 2DOF VIVPEH. Figure 19, Figure 20 and Figure 21 reveal the effects of the mechanical parameters (*m*_1_, *m*_2_, *k*_1_, *k*_2_, *c*_1_, *c*_2_) on the “lock-in” regions. The two boundary wind speeds of lock-in region I and II in each state were selected as the ordinate. At the same time, the same lock-in region was filled with solid color, so that the lock-in region is shown as a colorful ribbon. The difference between the upper and lower bounds of each ribbon represents the width of the lock-in region under that condition. Therefore, these three figures show the changing trend of the boundary and width of the two lock-in regions under the change in mechanical parameters.

Figure 19a,b show the effects of lumped mass *m*_1_ and *m*_2_ on the formation of the two “lock-in” regions, respectively. It is obvious to see that the effects of *m*_1_ and *m*_2_ are different. With the increase in *m*_1_, the boundaries of region I and II both decrease, and the upper boundary of region II changes most dramatically. This results in a rapid reduction in the bandwidth of region II. While the bandwidth of region I decreases at a glacial pace, in contrast, the influence brought by the increase in *m*_2_ has a more significant effect on region I rather than II. Therefore, we can conclude that regions I and II are, respectively, more sensitive to *m*_2_ and *m*_1_.

Figure 20a,b show the influences of mechanical damping *c*_1_ and *c*_2_. The mechanical damping coefficient *c*_1_ has a mild effect on the locking region: the bandwidth and position of both region I and II almost only slightly change in response to the change in *c*_1_. Unlike *c*_1_, increasing *c*_2_ lifts up the two “lock-in” regions, leading to an increase in the cut-in wind speeds. This can be explained by the instability theory of self-excited oscillation. The VIVPEH starts to vibrate when the effective linear damping coefficient becomes negative. Increasing *c*_2_ raises the threshold to turn the effective linear damping coefficient from positive to negative.

The influences of stiffnesses *k*_1_ and *k*_2_ on the two locking regions are demonstrated in Figure 21a,b. Compared with the results shown in Figure 19 and Figure 20, it can be seen that the influences of stiffnesses on the two lock-in region are much more profound. In particular, any increase in either *k*_1_ or *k*_2_ results in the upward movement and enlargement of region II (the blue and cyan-colored areas in Figure 21. However, regarding region I, *k*_1_ and *k*_2_ show completely different influences: increasing *k*_2_ widens region I, while *k*_1_ does the opposite.

**Figure 22:**
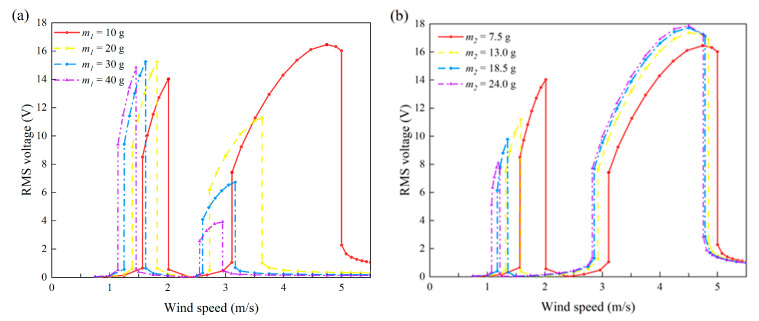
Effects of masses on the RMS voltage of the 2DOF VIVPEH: (**a**) *m*_1_; (**b**) *m*_2_.

**Figure 23:**
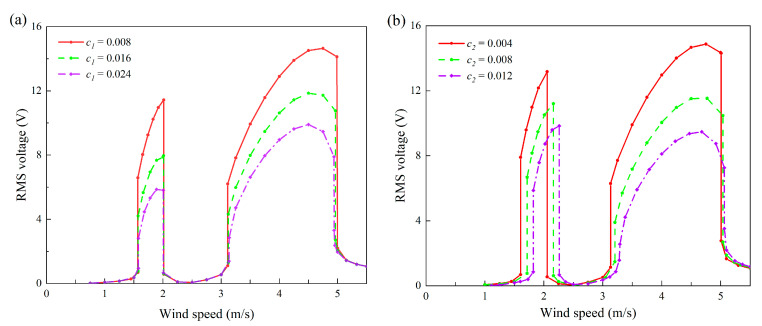
Effects of system damping on the RMS voltage of the 2DOF VIVPEH: (**a**) *c*_1_; (**b**) *c*_2_.

**Figure 24:**
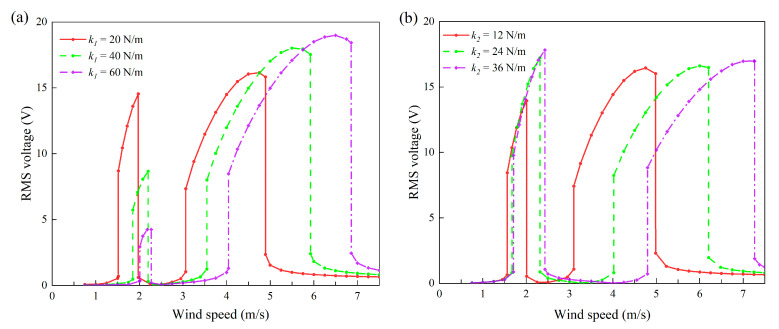
Effects of stiffnesses on the RMS voltage of the 2DOF VIVPEH: (**a**) *k*_1_; (**b**) *k*_2_.

In addition to the bandwidths and boundaries, the voltage amplitudes within the two lock-in regions were also of great interest to us. Hence, some mechanical parameters were selected equidistantly to draw the root-mean-square voltage diagram under the change in wind speed. In Figure 22, Figure 23 and Figure 24, except for the mechanical parameters indicated in the legend, the other parameters are consistent with Table 1; that is to say, the load resistance was always kept at 1 MΩ.

As can be seen from Figure 12, with the increase in the lumped mass *m*_1_ and *m*_2_, the two convexes on the voltage response curve form due to the lock-in regions gradually moving to the left, which is consistent with the results in Figure 19. Meanwhile, the mass variation also causes a change in the output voltage. However, the change in *m*_1_ and *m*_2_ brings different consequences to the different lock-in regions. With the increase in *m*_1_, the convex height of region I slightly increases, while that of region II significantly decreases. In contrast, increasing *m*_2_ leads to a significant decrease in the convex height of region I, with a slight increase in the convex height of region II. Therefore, from the voltage amplitude perspective, the previous conclusion that regions I and II are, respectively, more sensitive to *m*_2_ and *m*_1_ is still valid.

Figure 20 unveils the mechanical damping effects on the bandwidths and positions of the two lock-in regions. Figure 23 intends to reveal the influence of mechanical damping on the voltage output amplitude. Putting aside the bandwidth and position which have been discussed before, one can notice that the increase in damping leads to a decrease in the output RMS voltage. This is consistent with the general cognition: that is, the increase in damping will inevitably weaken the structural vibration and thus reduce the output from the VIVPEH.

We can figure out the influences of mechanical stiffnesses on the output characteristics of the VIVPEH in Figure 24. The effects on the bandwidth and position revealed by the several example cases in Figure 24 are in agreement with the conclusions obtained based on Figure 21. Regarding the voltage amplitude, the increase in *k*_1_ leads to a significant decrease in the RMS voltage in region I, but a slight increase in the RMS voltage in region II. In contrast, the increase in *k*_2_ brings an increase in the RMS voltage in region I, while the RMS voltage in region II is almost unaffected. The reasons for these phenomena are as follows: on the one hand, the increase in mechanical stiffness makes it more difficult to excite the vibration, which is manifested as the increase in the cut-in wind speed. On the other hand, the first- and second-order modes of the 2DOF VIVPEH have different sensitivities to the mechanical stiffnesses *k*_1_ and *k*_2_. That is why increasing *k*_1_ and *k*_2_ causes the system to take different dynamic behavior changes.

## 5. Conclusions

This paper has presented the study of a 2DOF VIVPEH for small-scale wind energy harvesting. Based on the traditional model of a 1DOF VIVPEH, the electromechanical governing equations of the 2DOF VIVPEH were established. The aerodynamic force was represented by a wake oscillator model with some empirically determined parameters. Subsequently, the dynamics and especially the energy harvesting performance of the 2DOF VIVPEH were investigated using numerical simulations. Finally, a parameter study was carried out to figure out the influence of the system parameters on the aerodynamics and energy harvesting performance of the 2DOF VIVPEH. The key conclusions from the above analyses are summarized below:

(1) The 2DOF VIVPEH has two “lock-in” regions, which is beneficial for broadband wind energy harvesting. In general, the bandwidth of region I is smaller than that of region II. Region I and region II correspond to the first- and second-order resonances, respectively.
(2) The optimal load resistance corresponding to the maximum output power is almost the same within the same lock-in region. However, the optimal load resistance corresponding to region II is smaller than that corresponding to region I. This is because different vibration modes are activated in the different lock-in regions.
(3) To reduce the cut-in wind speed, one can increase *m*_1_ and *m*_2_. However, considering the voltage output within the lock-in regions, *m*_1_ and *m*_2_ cannot be arbitrarily tuned. Regarding *c*_1_ and *c*_2_, they should be reduced as much as possible to decrease the cut-in wind speed and enhance the voltage output. Certain environmental factors should be taken into account in selecting *k*_1_ and *k*_2_. For example, when the ambient wind speed is low (2 m/s), one can select a relatively large *k*_2_. When the ambient wind speed is high (5 m/s), it is suggested to increase *k*_1_ properly.

## Figures and Tables

**Table 1:** Parameters of 2DOF VIVPEH.

Mechanical Parameters		Aerodynamic Parameters	
Effective mass *m*_1_ (g)	10	Air density, *ρ* (kg × m^−3^)	1.204
Effective mass *m*_2_ (g)	7.5	Bluff body height, *L* (m)	0.203
Effective stiffness *k*_1_ (N × m^−1^)	21.985	Cross flow dimension, *D* (m)	0.0396
Effective stiffness *k*_2_ (N × m^−1^)	12.15	Amplitude of the fluctuating lift force coefficient, *C*_*L*0_	0.3
Effective damping *c*_1_ (N × s/m)	0.0041		
Effective damping *c*_2_ (N × s/m)	0.0026	Mean drag coefficient, *C_D_*	2
Electromechanical coupling *θ* (μN × V^−1^)	39.657	Modal constants, *λ*	0.3
Capacitance, *C_p_* (nF)	30.78	Modal constants, *A*	12
Resistive load, *R_L_* (MΩ)	1	—	—

**Table 2:** RMS voltage at several typical wind speeds.

Wind Speed	RMS Voltage	Wind Speed	RMS Voltage
(m/s)	(V)	(m/s)	(V)
1.565	0.663	1.566	8.511
2.015	14.038	2.016	0.563
3.108	1.069	3.109	7.422
5.000	16.023	5.001	0.6627
